# Monitoring Free‐Living Honeybee Colonies in Germany: Insights Into Habitat Preferences, Survival Rates, and Citizen Science Reliability

**DOI:** 10.1002/ece3.71469

**Published:** 2025-06-05

**Authors:** Benjamin Rutschmann, Felix Remter, Sebastian Roth

**Affiliations:** ^1^ BEEtree‐Monitor Munich Germany; ^2^ Department of Animal Ecology and Tropical Biology, Biocenter University of Würzburg Würzburg Germany; ^3^ Agroecology and Environment, Agroscope Zurich Switzerland; ^4^ Rachel Carson Center for Environmental Humanities, LMU Munich Munich Germany; ^5^ Department of Science Technology and Society (STS) at the TUM School of Social Sciences and Technology, TU Munich Munich Germany

**Keywords:** *Apis mellifera*, liminal species, multi‐species studies, urban ecology, wild pollinators, wildlife conservation, wildlife monitoring, wild‐living honeybees

## Abstract

Our understanding of the western honeybee ( *Apis mellifera* ) predominantly stems from studies conducted within beekeeping environments, leaving the presence and characteristics of honeybees outside managed settings largely unexplored. In this study, we examined the habitats, nesting sites, and survival rates of free‐living colonies through personal monitoring of nest sites in Munich (*N* = 107) and the coordination of Citizen Science monitoring across Germany (*N* = 423). Within 7 years, we collected 2555 observations on 530 nest sites from 311 participants, including the authors. Overall, we found that 31% of the occupied nest sites were in buildings and 63% in mature trees, with clear preferences for specific tree species. Nesting preferences differed between urban, rural, and forested areas. On average, only 12% of the personally monitored colonies in Munich survived annually, a figure that aligns well with other published studies in Germany but contrasts sharply with the significantly higher survival rates resulting from Citizen Science reports (29%)—a discrepancy likely driven by certain reporting biases. We found that Citizen Science yielded significantly fewer updates per colony, underreported abandoned sites, and that 46% of overwintering reports overlapped with the swarming season and had to be excluded. To gain reliable survival data in Citizen Science projects, consistency and timing of reports need particular attention and regional swarming should be monitored as well. This study enhances our understanding of the ecological dynamics, liminal state, and conservation needs of free‐living honeybee cohorts, addresses potential Citizen Science monitoring biases, and suggests standardized data collection protocols for future monitoring projects. The preservation of mature trees with suitable cavities, as well as the provision of additional nesting sites, is key for sustaining free‐living honeybee cohorts and should be integrated into conservation strategies, urban planning, and forest management.

## Graphical abstract

In this study we examined the habitats, nesting sites, and survival rates of free‐living colonies through personal monitoring of nest sites in Munich (*N* = 107) and the coordination of Citizen Science monitoring across Germany (*N* = 423). On average, only 12% of the personally monitored colonies in Munich survived annually, a figure that aligns well with other published studies in Germany but contrasts sharply with the significantly higher survival rates resulting from Citizen Science reports (29%)—a discrepancy likely driven by certain reporting biases. This study enhances our understanding of the ecological dynamics, liminal state, and conservation needs of free‐living honeybee cohorts, addresses potential Citizen Science monitoring biases, and suggests standardized data collection protocols for future monitoring projects.

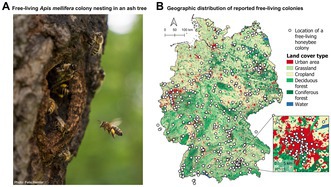

## ABSTRAKT

Unser Verständnis der Westlichen Honigbiene (*Apis mellifera*) basiert überwiegend auf Forschung, die unter imkerlichen Bedingungen durchgeführt wurde, während das Vorkommen und die Lebensweise von Honigbienen abseits menschlicher Haltungsformen weitgehend unerforscht sind. Für die vorliegende Studie wurden die Lebensräume, Nistplätze und Überlebensraten freilebender Völker durch eigene Beobachtungen in München (N = 107) sowie durch die Koordination eines Citizen‐Science‐Monitorings in ganz Deutschland (N = 423) untersucht. Über sieben Jahre sammelten 311 Mitwirkende, einschließlich der Autoren, insgesamt 2555 Beobachtungen an 530 Standorten. Die Nistplätze befinden sich zu 31% in Gebäuden und zu 63% in alten Bäumen, wobei eine deutliche Präferenz für bestimmte Baumarten erkennbar ist. Die Nistpräferenzen unterscheiden sich zwischen urbanen, ländlichen und bewaldeten Gebieten. Im Durchschnitt überleben jährlich nur 12% der durch die Autoren in München beobachteten Völker. Während andere Studien vergleichbar geringe Überlebensraten in Deutschland bestätigen, weichen die deutlich höheren Überlebensraten unserer Citizen‐Science‐Meldungen (29%) davon ab – ein Unterschied, der durch Meldebiases verursacht wird. Wir stellten fest, dass im Rahmen von Citizen Science signifikant weniger Statusmeldungen pro Volk gemacht und Standorte, an denen Völker gestorben sind, seltener gemeldet wurden. Ausserdem überlappten 46% der Überwinterungsmeldungen mit der Schwarmzeit und mussten daher ausgeschlossen werden. Deshalb sind Konsistenz und Zeitpunkt der Beobachtung zentral, um verlässliche Überlebensdaten zu sammeln; zudem sollte die regionale Schwarmzeit berücksichtigt werden. Die vorliegende Studie soll unser Verständnis der Ökologie, des liminalen Status und der Schutzbedürftigkeit freilebender Honigbienenvölker verbessern, thematisiert potenzielle Verzerrungen in Citizen‐Science‐Daten und schlägt ein standardisiertes Monitoringprotokoll für zukünftige Projekte vor. Der Erhalt alter Bäume mit geeigneten Höhlen sowie die Bereitstellung zusätzlicher Nistplätze sind entscheidend für den Fortbestand freilebender Honigbienenvölker und sollten in Naturschutzstrategien, Stadtplanung und Forstwirtschaft Berücksichtigung finden.

## 1 Introduction

The western honeybee ( *Apis mellifera* ) holds a significant place in the European entomofauna, facilitating the reproduction and genetic diversity of countless plant species, including many agricultural crops (Garibaldi et al. 2013; Breeze et al. 2014; Hung et al. 2018). As a cavity‐dwelling species, *Apis mellifera* is adapted to live in forests, with tree hollows serving as its original nesting sites (Crane 1999; Ruttner 1988; Zander 1949). Although the species has been used for honey harvesting since the Neolithic period (Crane 1999) and plays a key role in modern commercial pollination services, it has undergone relatively little selective breeding compared to other similarly intensively managed animals (Oxley and Oldroyd 2010). Nevertheless, among researchers and beekeepers alike, *Apis mellifera* tends to be perceived solely as a domesticated animal, found and researched under managed conditions. Consequently, most of our comprehensive understanding of the western honeybee as a species, its behavior, and its ecology predominantly stems from research conducted with colonies under beekeeping conditions, while wild honeybee populations have been neglected in the modern apidological tradition (Stoeckhert 1954; Kohl and Rutschmann 2018; Seeley 2019; Requier et al. 2019). The introduction of the parasite *Varroa destructor* (Anderson and Trueman 2000) to Europe in the 1970s intensified this oversight, as the subsequent regular miticide treatment suggested that only human‐managed colonies could survive (Rosenkranz et al. 2010; Meixner et al. 2015).

Yet, this belies the fact that the western honeybee is also present outside the realms of beekeeping and human husbandry (Grindrod and Martin 2021; Kohl and Rutschmann 2018; Visick and Ratnieks 2023) and how little is known about the formation and dynamics of this cohort. Currently, stable wild honeybee populations within their original range are known to exist in Africa and the Southern Ural, and outside their original range in the Americas and Australia (Schneider and Blyther 1988; Moritz et al. 2007; Ilyasov et al. 2015; Ratnieks et al. 1991; Guzman‐Novoa et al. 2024; Seeley 2007; Bozek et al. 2018; Oldroyd et al. 1997; Chapman et al. 2008). Studies on these populations usually focus on habitats where managed and non‐managed colonies are relatively separated, or where the density of wild colonies matches or surpasses that of managed colonies (Jaffé et al. 2010; Visick and Ratnieks 2023). The news about stable populations of western honeybee colonies outside beekeeping raised the interest of beekeepers in how to escape treatment (Seeley 2019; Remter 2021a) and spurred considerable research in this field. In Europe, free‐living colonies have been documented in various environments, ranging from forests to electric power poles in agricultural landscapes and rural and urban areas (Oleksa et al. 2013; Kohl and Rutschmann 2018; Requier et al. 2020; Oberreiter et al. 2021; Kohl et al. 2022; Rutschmann et al. 2022; Hassett et al. 2018; Browne et al. 2020; Moro et al. 2021; Bila Dubaić et al. 2021; Lang et al. 2022; Visick and Ratnieks 2024; Cordillot 2024). However, the situation in Europe is different due to its fragmented landscape (Ibisch et al. 2016; Lesiv et al. 2019) and the high density of managed colonies (Phiri et al. 2022; Jones 2004), which means there is no spatial and genetic barrier between managed and free‐living colonies in most parts. The primary differences between free‐living colonies and managed ones lie in their nest site ecology and their *modus vivendi*. Consequently, we propose referring to them as cohorts of local honeybee populations that are neither *fully wild* nor *domesticated* but exist in a *liminal state*.

To understand the free‐living honeybee cohort more completely, it is crucial to examine not only their nesting sites but also the survival rates, which are critical for assessing their genetic contribution to the local honeybee population. However, most of the studies on free‐living honeybees in Europe have not systematically monitored individual colony survival and have instead reported on nesting sites without comprehensive knowledge of the life histories of individual colonies—exceptions include Rutschmann et al. (2022), Kohl et al. (2022), Lang et al. (2022) and Cordillot (2024). Due to their hidden locations in cavities high above the ground, free‐living colonies lead secretive lives (Kohl and Rutschmann 2018; Remter 2021b): finding them and repeatedly collecting data in numbers high enough for statistical inference is very time‐consuming and requires specialized skills and equipment (Kohl and Rutschmann 2018; Kohl et al. 2022). One approach that has garnered significant attention in the study of wild animals and biodiversity monitoring is the utilization of Citizen Science (Pocock et al. 2018; Fraisl et al. 2022; Koffler et al. 2021; Weissmann et al. 2023). Citizen Science offers the advantage of enlisting the help of many individuals who, in our case, shared the task of finding and monitoring colonies, thereby extending the geographic and temporal scope of the research beyond what researchers could achieve alone (Henneken et al. 2012; Lesiv et al. 2019; Hsing et al. 2022). Also, although previous studies have acknowledged the importance of Citizen Science in data collection (Moro et al. 2021, 2024; Bila Dubaić et al. 2021), none have yet investigated the quality of the data generated by Citizen Science and how to validate such reports.

With the BEEtree‐Monitor, we developed a web‐based monitoring scheme to study the habitats, nesting sites, and life histories of free‐living honeybee colonies. Over a span of 7 years (2016–2023), we collected various parameters of 530 nest sites together with longitudinal occupation data: 107 nest sites monitored by ourselves in the Munich region and 423 by citizen scientists mostly in Germany. Besides the main analysis, we compare these two approaches to evaluate the validity and potential biases of Citizen Science reports and methodology. Through this study, we aim to provide a broader understanding of honeybees as a species that also exists outside human husbandry, provide insights into conservation strategies to support them, and offer guidelines to leverage future Citizen Science projects for effective monitoring of free‐living honeybee colonies.

## 2 Methods

### 2.1 Data Collection and Data Curation

**FIGURE 1:**
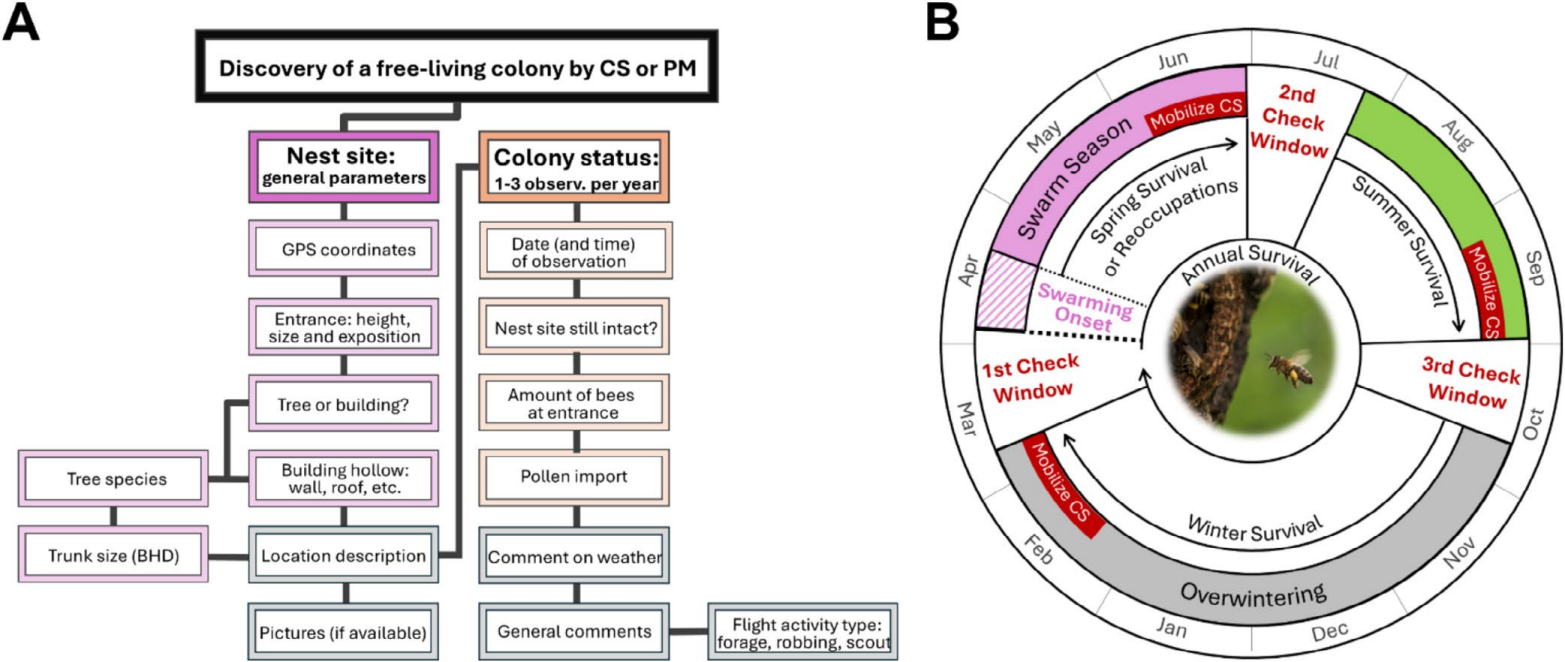
(A) Monitoring protocol for Citizen Science (CS) and personal monitoring (PM) with steps for recording nest site parameters (violet) and colony status (orange). Optional information like location descriptions, weather comments, general comments, and comments on flight activity type (sometimes including pictures and videos) helps validating the data (indicated in gray). (B) The annual monitoring schedule for free‐living honeybee colonies outlines three critical observation windows: 1st check window (before the onset of the swarming season in late March and April), 2nd check window (post‐swarming season in July), and 3rd check window (in autumn). These checks focus on different survival metrics: Winter survival, spring survival and reoccupations and summer survival, respectively. Citizen scientists (CS) mobilization should be done before each check window to ensure timely data collection. The yearly onset of swarming shifts and must be monitored closely to ensure accurate data on colony survival. The inlet picture shows pollen import into the colony an indication for brood production (photo: Felix Remter).

The monitoring and data collection process for this study was implemented through a combination of personal monitoring (PM) and reports by volunteering supporters with highly diverging skill levels and knowledge sets (citizen science monitoring, CS). Leveraging our own experiences in surveying free‐living honeybee colonies and third‐party reports, we developed an advanced monitoring scheme designed for our target groups. In 2018, we constructed an online platform specifically tailored for Citizen Science monitoring, launched as a website (BEEtree‐Monitor; www.beetrees.org; see Supporting Information for further information). The CS recruiting was facilitated through social media outreach, presence in public media, and beekeeping journals. The community was maintained via regular newsletters with guiding and motivating information. To enable as many volunteers as possible without offering a special training, our protocol focused on location, easily measurable nest site parameters, and continuous, repeated observations with specific date, time, and focus (Figure 1A). Precise GPS coordinates allowed us to investigate the nesting and foraging habitats of the colonies, and the nest site parameters were used to analyze the swarms' preferences for different nest types such as hollows in building structures (e.g., chimneys, window blind boxes or facade compound insulations) or hollows in different tree species. Additionally, we sought information on entrance directions and height, as well as the trunk diameter for trees. The online platform also featured an open‐text/commentary field for participants to provide other relevant details observed.

Both PM and CS follow the same basic protocol (Figure 1A). Generally, our approach involved providing participants with protocols and guidance to for example, prioritize pollen import observation (indicating brood production, marking the colony as active). Accessible explanations in the online form and regular emails encouraged detailed reporting during key observation periods.

In total only 36 nesting sites were found by the authors (and 494 by citizen scientists), however 107 locations were actively monitored by us (and 423 by citizen scientists). Our primary analysis focused on colonies located in Germany and several nearby Central European countries, including Switzerland (*N* = 9), Austria (*N* = 3), Czechia (*N* = 2), and Luxembourg (*N* = 3). To mitigate the influence of markedly different environmental or population conditions, we excluded reports of ten free‐living colonies from geographically distant countries like France, the UK, Italy, Spain, Norway, and Ukraine.

### 2.2 Analysis of Nesting and Foraging Habitats

To classify the nesting and foraging habitats of the reported free‐living colonies, we imported the coordinates of each colony's location into QGIS version 3.16.2 (QGIS Development Team 2020) and performed intersections (using point layers for nesting habitats and 2 km buffers for foraging habitats) with the land cover classes from the CORINE Land Cover map 2018 (European Environment Agency 2018). We grouped different land cover types together and quantified the proportional contributions of five major land cover types: urban areas, cropland, grassland, deciduous forest, and coniferous forest (see Supporting Information for further details). For part of the analysis, these land cover types were further grouped into the three categories: Urban (urban areas), rural (cropland and grassland), and forest (deciduous and coniferous forests). In the case of the foraging habitat, we chose a radius of 2 km as approximately 80% of honeybee foraging occurs within this distance (Rutschmann et al. 2023). Moreover, the landscape within the 2 km scale has been shown to measurably influence honeybee colony performance, affecting factors such as foraging rate, colony growth, and winter survival (Steffan‐Dewenter and Kuhn 2003; Sponsler and Johnson 2015; Rutschmann et al. 2022, 2023).

### 2.3 Observation Scheme for Colony Survival Statistics

We defined survival as the instance of a cavity being occupied from summer (during or after the swarming season) until the following spring, before the next swarming season commenced (Figure 1B). One valid report of an active colony in spring before the start of the swarming season (1st check) is proof of overwintering survival. Additionally, we implemented and encouraged participants to conduct one or two more annual checks: after the end of swarming (2nd check) and in Autumn (3rd check). Post‐swarming checks at all known cavities (including the recently unoccupied ones) served to find the new founder colonies for further monitoring, while the Autumn check (3rd check) detects summer deaths (we attributed these as perished to the survival statistics of the following year). For assessing overwintering, observations typically take place in March or early April, where it is crucial that weather conditions are suitable for honeybee foraging (e.g., no rain and temperatures above 12°C. [Kevan and Baker 1983)] but before swarming and therefore reoccupation of cavities. Consequently, observations without suitable weather conditions for honeybee foraging were excluded. We only considered primary data for our analysis; oral reports or in retrospect reports (of colonies living for “several years” in the same cavity) were considered hearsay and excluded. We used the same data analysis pipeline for both PM and CS, and in most cases, it was not known whether the colony was monitored by the authors or by citizen scientists. Destroyed nesting sites were also not considered in the survival statistics for the year of destruction. In addition to annual survival rates, we also calculated spring, summer, and winter survival rates for comparison with other studies (see Supporting Information for further information).

A potential pitfall of reporting mere flight activity at the entrance is that certain behaviors such as robbing, or the presence of scout bees may be erroneously interpreted as signs of a living colony by less experienced observers. Therefore, pollen import into a colony was used as an indicator of brood production, hence designating it as an alive colony. Under certain conditions, even in the absence of visible pollen import, we still considered the colony to be alive. These criteria included:

1. *Observation of foraging flight patterns*: Colonies were classified as active if regular and/or directional flight activity was reported convincingly in the commentary section. These patterns are differentiated from non‐foraging activities specifically observed in PM:
  – Scout bees typically exhibit distinct behaviors such as taking time to land, thoroughly exploring the entrance before entering the cavity, performing slow orientation flights during departure, and might defend the entrance against other scouts when near swarming.
  – Robbing bees display violent interactions with defenders if attempting to rob a living colony. If emptying a perished colonies stock, robbers initially perform orientation flights akin to scouts but more hectic with a distinct “bouncing off” landing pattern. Landing bees are jumped at by exiting bees.
2. *Reliability of observer comments*: If the observer was suspected to credibly discern differences in flight patterns indicative of foraging rather than robbing or scouting due to a competent comment given, the colony was considered active.
3. *Frequency and timing of observations*: Multiple observations made at short intervals that consistently indicated foraging behavior, as opposed to scouting or robbing, supported the classification of a colony as active.

### 2.4 Exemplary Estimation of Colony Density

Estimating the density of free‐living honeybee colonies poses challenges due to the likelihood of substantial underreporting. To address this, we concentrated our density estimation efforts on the city of Munich (further details can be found in the Supporting Information).

### 2.5 Onset of Regional Swarming

In 2019, two of the authors (SR and FR) established a website across Germany and a hotline in Munich for the discovery of honeybee swarms and their potential capture. This enabled us to amass substantial data (*N* = 376) on the initiation of swarming activity in the years from 2019 to 2023 within the geographic extent of this study. These years also represent the period during which most of the nest sites presented in this study were found (*N* = 378 out of 530 colonies) and monitored (*N* = 307 out of 350 life history reports). For each year, the first reported swarm served as a conservative estimate of the commencement of the swarming season. The onset of swarming varied over the five‐year period. Specifically, swarming began on 17 April in 2019, 6 April in 2020, 8 May in 2021, 28 April in 2022, and 23 April in 2023. Hence, the time difference between the earliest and the latest recorded yearly swarming onset was more than 1 month (32 days), suggesting it should be taken into account when planning CS initiatives and survival analyses of free‐living honeybees, especially in years where swarming occurs unexpectedly early (e.g., year 2020 or 2024). A small subset of the free‐living colony reports in this study stem from years before 2019 in which we lacked empirical data on the beginning of the swarming season. Hence, we conservatively selected mid‐April as the presumed start of swarming for these years (Henneken et al. 2012).

### 2.6 Comparing Survival Rates of Free‐Living Honeybee Colonies

To analyze colony survival statistics across different years in Germany we compared results from CS and PM and included two published studies—Kohl et al. (2022) and Lang et al. (2022). To ascertain the influence of various predictors including monitoring types and year on the odds of colony survival, we compared several mixed‐effects logistic regression models (see Supporting Information for further details). These models were constructed using the “glmmTMB” package in R (Magnusson et al. 2017). The model selection process was guided by the Akaike Information Criterion (AIC) and the “emmeans” package (Lenth and Lenth 2018) was used for post hoc comparisons. Model predictions and confidence intervals were generated using the “ggeffects” package (Lüdecke 2018). Residuals of the models were inspected with “DHARMa” package (Hartig and Hartig 2017).

### 2.7 Statistical Analysis of Directional and Height Preferences in Cavities

To evaluate directional preferences of honeybees when selecting cavities, we recorded the entrance orientation of nesting sites occupied by free‐living colonies in the eight cardinal and intercardinal directions for both tree cavities and cavities in buildings and assessed whether the distribution of orientations was non‐random by employing the Rayleigh test for circular statistics using the Directional package in R (Tsagris et al. 2016). The height of cavity entrances in trees and building structures was investigated with a non‐parametric Mann–Whitney *U* test.

Further information on statistical analysis regarding the number of reports per colony, reported colony status, and timing of the reports can be found in the Supporting Information.

All statistical analyses were performed using R software (version 4.3.1; R Core Team 2016). For data wrangling and graphical representation of the results, we utilized “tidyverse”, “ggplot2”, “patchwork”, “see” and “ggpattern” (Wickham 2017, 2016; Pedersen 2020; Lüdecke et al. 2021; FC and Davis 2024).

## 3 Results

### 3.1 Nesting and Foraging Habitat

**FIGURE 2:**
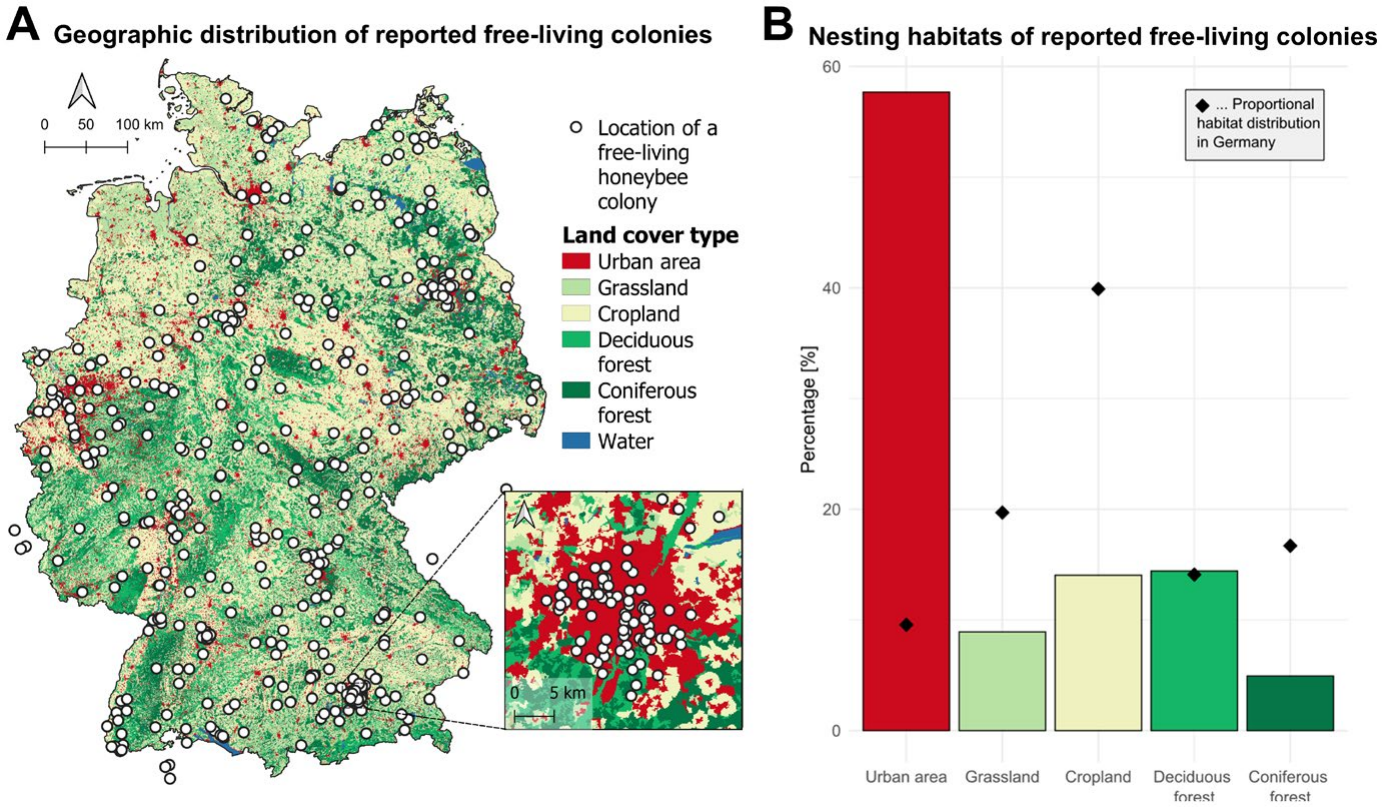
(A) Geographic distribution of the free‐living colonies reported, overlaid on the grouped main land cover types within Germany (CORINE Land Cover map 2018). The inlet shows the free‐living colonies in the Munich region (three colonies outside Germany are hidden by the inlet). (B) Proportional distribution of nesting habitats where free‐living colonies have been reported in comparison to the relative distribution of different land cover types across Germany depicted by black diamond symbols.

The 311 participants (including the authors) provided 2555 observations on 530 free‐living colonies (Figure 2A). We found 58% of the colonies were reported from urban areas with high human density, 14% from deciduous forest, 14% from cropland, 9% from grassland and 5% from coniferous forest (Figure 2B; available foraging habitats are shown in Supporting Information). The distribution of reported nesting habitats differed significantly from the proportional availability of land cover types in Germany (*χ* ^2^ = 99.81, df = 4, *p* < 0.001). Colonies were disproportionately more often reported from urban areas, equally often from deciduous forests, and less often from grassland, cropland, and coniferous forests relative to their availability in the landscape. However, these data likely reflect a combination of true nesting occurrence and varying detection probabilities, as colonies in urban areas are more readily discovered due to closer human proximity. Additionally, the high density of managed honeybee colonies in urban areas likely increases the rate of swarm escape and the number of newly founded free‐living colonies in these habitats, contributing further to a certain urban bias.

### 3.2 Nesting Characteristics

**FIGURE 3:**
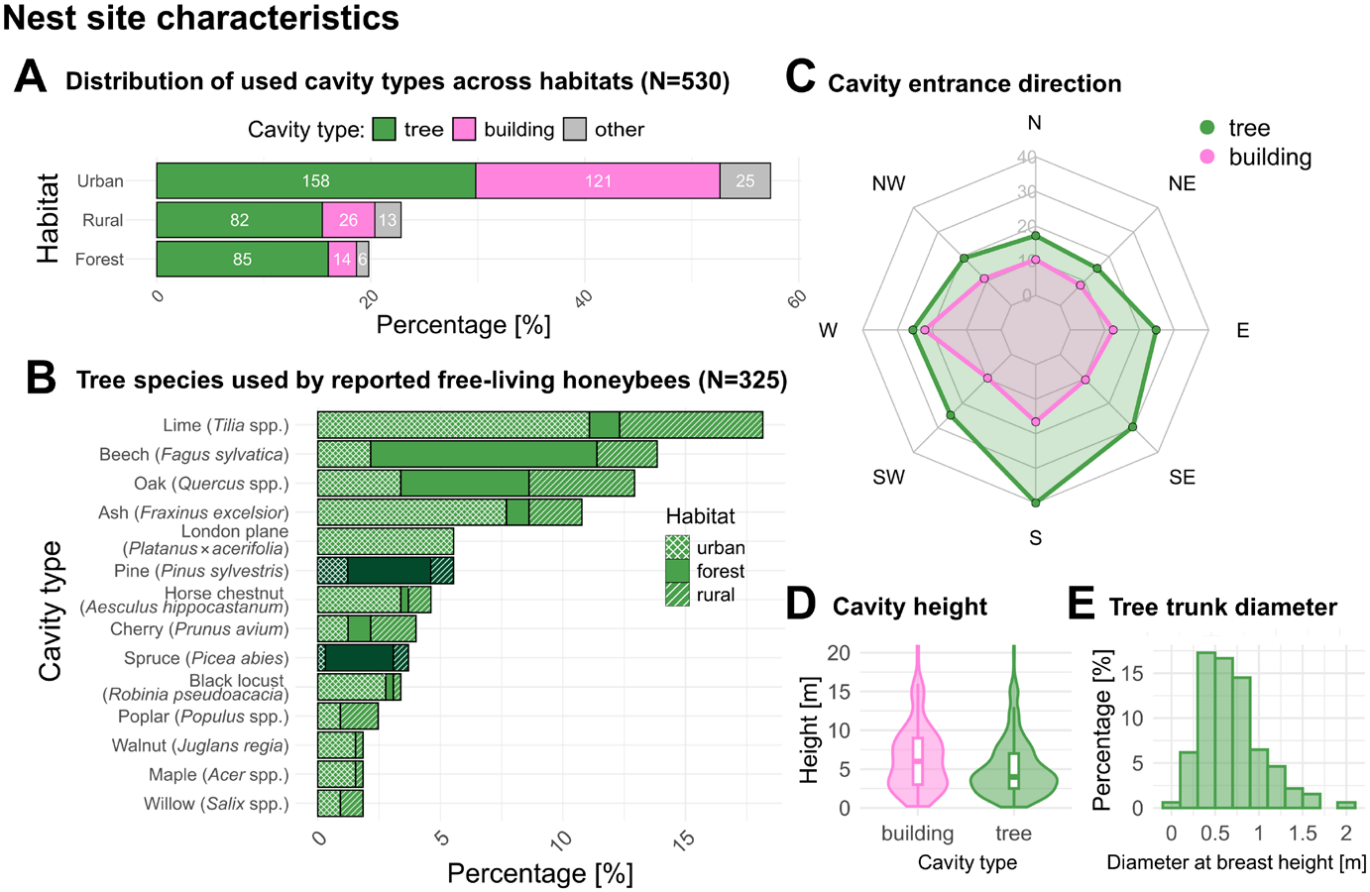
(A) Proportional distribution of nesting cavity types (tree, building or other) across the habitats. While the *x*‐axis gives the overall percentage, white numbers indicate the count (*N*) of trees, buildings, and other cavities. The category “other” includes 10 colonies for which no cavity type was reported. (B) Distribution of tree species used as nesting sites reported in this study. Pattern fills illustrate the distribution across different habitats and darker green fill represents coniferous tree species. (C) Preferred nesting entrance directions for cavities in trees and buildings. (D) Cavity height distribution: A violin plot with an embedded boxplot that demonstrates the range and distribution of the heights of cavities occupied. (E) Distribution of tree trunk diameters (measured at breast height) for trees that hosted honeybee colonies.

The ratio of tree cavities to nesting sites in buildings, as well as the proportions of reported tree species, differed between urban, rural, and forest habitats. For the reported colonies in urban areas (57%, *N* = 304), tree cavities comprised 52% of all cavities, while building cavities accounted for 40% (other cavity types: 8%; Figure 3A). In rural areas (23%, *N* = 121), tree cavities were again dominating (68%), followed by cavities in buildings (21%). In forest areas (20%, *N* = 105), tree cavities accounted for 81% of all nesting sites, while cavities in buildings represented 13%. Looking at the whole dataset 63% (*N* = 325) of the colonies were found in trees, 31% (*N* = 161) in building structures, and the remaining colonies (*N* = 34) in other types such as rock crevices (*N* = 3) and open nesting (*N* = 15). Among tree species, Lime (*Tilia* spp.) was the most frequently occupied (*N* = 59; 18%), followed by beech ( *Fagus sylvatica* , *N* = 45; 14%), oak (*Quercus* spp., *N* = 42; 13%) and ash ( *Fraxinus excelsior* , *N* = 35; 11%) (Figure 3B). However, the distribution of these species differed across habitats (Figure 3B). Especially in urban areas, the diversity of tree species used by bees is much higher, with lime (23%), ash ( *Fraxinus excelsior* , 16%), plane ( *Platanus × acerifolia* , 11%), and horse chestnut ( *Aesculus hippocastanum* , 7%) being the most frequently occupied.

Our study uncovered clear patterns in common flight entrance directions of nest sites used. For colonies situated in tree cavities, the observed frequency distribution across the eight cardinal and intercardinal directions was significantly non‐uniform (Rayleigh Test Statistic = 20.23, Bootstrap *p*‐value = 0.0010; *N* = 285). We found a significant preference for southern directions, with the highest frequency observed in the South direction (*N* = 58 or 20%; Figure 3C). Similarly, for colonies located in buildings, the distribution was tested to be marginally non‐uniform (Rayleigh Test Statistic = 4.85, Bootstrap *p*‐value = 0.079; *N* = 146). Unlike tree cavities, cavities in human‐built structures showed no clear directional preferences, although the West (*N* = 32 or 22%) and South (*N* = 24 or 16%) directions were observed most frequently (Figure 3C).

Our observations indicate that honeybee swarms predominantly choose nesting sites far from the ground (mean and median entrance height: 5.7 and 4.5 m; Figure 3D). There was a statistically significant difference in the height of the cavity entrance in trees and in buildings (Mann–Whitney *U* test *W* = 13,482, *Z* = −6.49, *p* < 0.001). The entrance heights of cavities occupied in trees ranged from 0.1 to 30 m, with a median of 4 m. In contrast, the heights of cavity entrances in man‐made structures ranged from 0.2 to 40 m, with a median of 6 m.

Additionally, we found that the diameter at breast height (DBH) of trees harboring free‐living honeybee colonies was on average 0.64 m (median: 0.69 m, Figure 3E), suggesting that colonies are dependent on trees with a substantial trunk diameter.

### 3.3 Occupation Rates and Colony Density Across Seasons in Munich

We investigated cavity occupation rates in the city of Munich during three distinct seasonal periods (spring, summer and autumn, see Supporting Information for further details). Multiplying the occupation rates by the density of 0.58 cavities per square kilometer that we know of in Munich (92 nest sites on an area of 160 km^2^, see Supporting Information), we estimated the minimum density of free‐living honeybee colonies in Munich to be approximately 0.06 colonies per square kilometer in spring, 0.42 in summer, and 0.28 in fall. It is important to note that the reported densities should be viewed as minimum estimates, given the likelihood that a significant number of colonies remain undetected and unreported. Based on these occupation rates, we infer that the number of colonies during summer is roughly seven‐fold higher compared to spring. Conversely, the number of colonies in fall is approximately 33% lower than in summer.

### 3.4 Survival Rates and the Impact of Monitoring Type and Year

**FIGURE 4:**
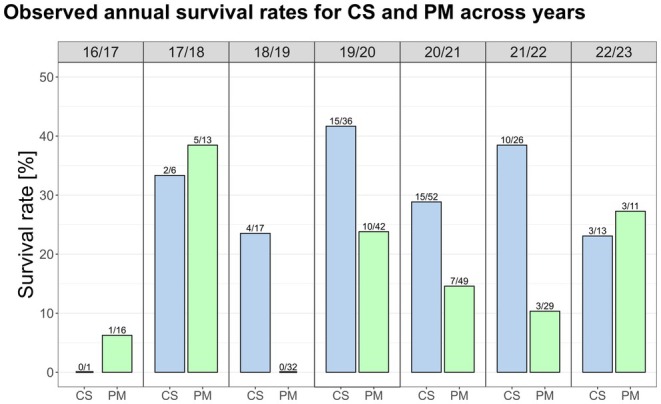
Comparison of observed annual colony survival rates from Citizen Science monitoring (CS, light blue) and personal monitoring in the Munich region (PM, light green) across the years. Numbers indicate the colonies that survived compared to the total reported.

We analyzed the life histories of 343 free‐living honeybee colonies over the period from 2016 to 2023. Of these, 151 survival reports were provided by citizen scientists, while 192 were personally observed in Munich. It is important to note that a single colony could have had multiple survival reports, as it was monitored across different years. In most years, survival rates reported by citizen scientists were higher than those observed through personal monitoring (Figure 4). Notably, in the spring of 2019, all PM colonies (*N* = 32) perished, while 4 out of 17 colonies monitored through CS were reported as having survived. We observed a similar pattern when analyzing seasonal survival rates. For PM, survival rates were 87% in spring (*N* = 23), 82% in summer (*N* = 173), and dropped to 21% in winter (*N* = 139). In contrast, CS survival rates were consistently higher, with 100% in spring (*N* = 24), 93% in summer (*N* = 180), and 46% in winter (*N* = 98; see Supporting Information for more information).

**FIGURE 5:**
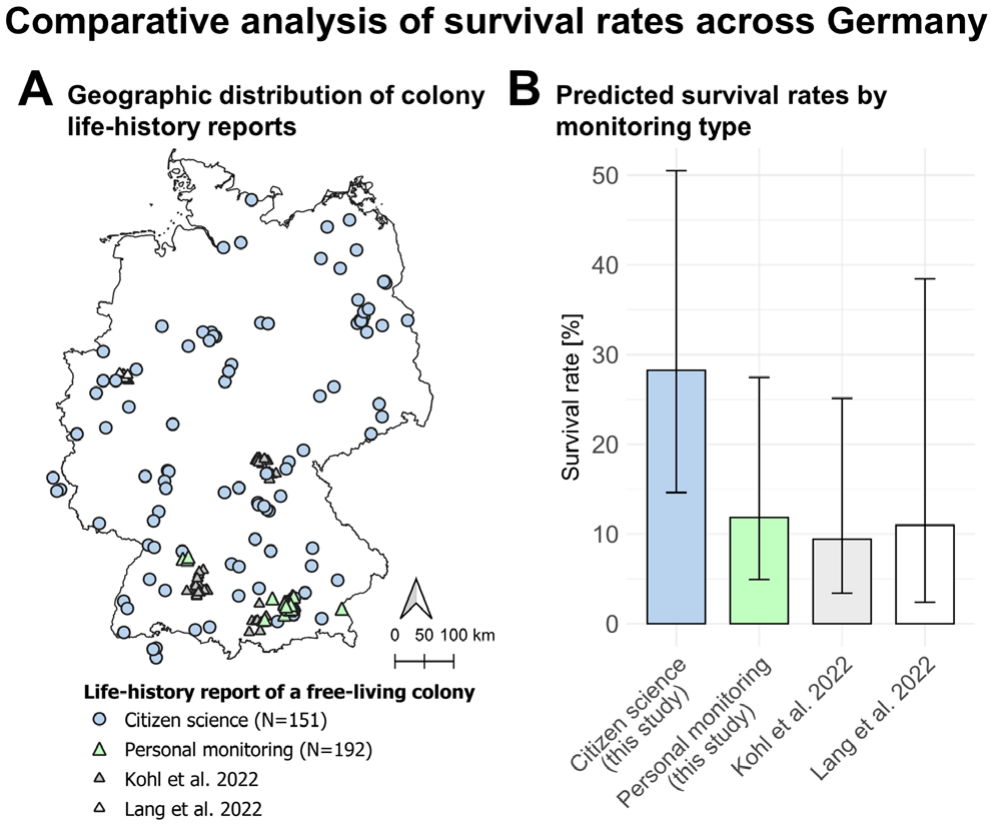
(A) Geographic distribution of life‐history reports of free‐living colonies from this study (CS and PM) and two published datasets –Kohl et al. (2022) and Lang et al. (2022). (B) Model estimates and 95% confidence intervals for average annual colony survival rates for the different monitoring types and studies.

Additionally, we consulted data from two published studies to determine whether the differences between CS and PM were likely due to biases in CS or actual differences. Kohl et al. (2022) looked at 112 colonies in German managed forest landscapes from 2017 to 2021, while Lang et al. (2022) investigated 30 colonies in Dortmund, Germany from 2018 to 2022 (Figure 5A). We selected a model including “year” as a fixed effect alongside the “type of monitoring” [CS, PM, Kohl et al. 2022 and Lang et al. 2022] and added “colony id” as a random effect to account for repeated measures on the same nest ids across years. The likelihood ratio test (LRT) results from the glmmTMB model indicated that both “monitoring type” (χ^2^ = 15.92, df = 3, *p* = 0.001) and “year” (χ^2^ = 18.28, df = 6, *p* = 0.006) significantly contributed to the model. The estimated probability of survival was notably higher in CS (29%) than in PM (12%, *p* = 0.005) and in Kohl et al. (2022) (13%, *p* = 0.02), but not significantly different from Lang et al. (11%, 2022) (*p* = 0.32; probably due to the small number of colonies) (Figure 5B). While minor variations may arise from regional or habitat conditions, the significant discrepancies observed are likely due to underreporting of abandoned sites in CS compared to systematic surveys by experts (see section 3.5).

### 3.5 Biases With Different Monitoring Types

**FIGURE 6:**
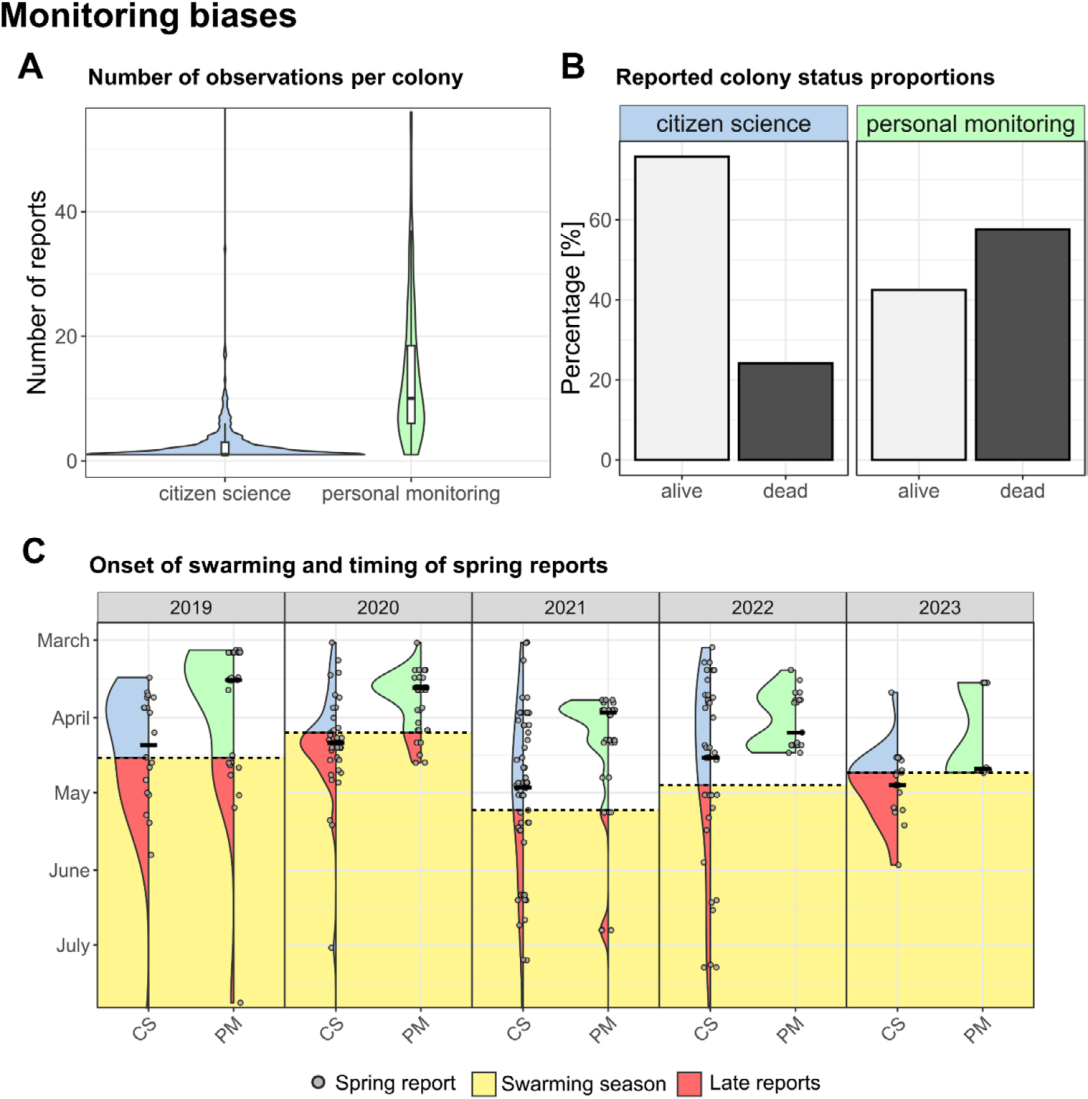
Analysis of reporting biases between Citizen Science monitoring (CS, light blue) and personal monitoring in the Munich region (PM, light green). (A) Violin plot with included boxplot illustrating the distribution of the number of observations per colony for the whole monitoring timespan. (B) Proportional representation of reported colony statuses (“alive” in light gray, “dead” in dark gray) for CS and PM. (C) Temporal variation in colony statuses reported post‐winter for the two different monitoring approaches in relation to the swarm season (in yellow) for the years 2019–2023. The dashed line marks the first observed swarm for each year, after which spring reports on colony survival status may not be reliable anymore. Bold horizontal lines indicate median values of the timing of CS and PM reports.

Our dataset comprised 423 nest sites with 1064 Citizen Science (CS) observations and 107 nest sites with 1491 personal monitoring (PM) observations. We noted a significantly lower number of CS reports per colony compared to PM (Wilcoxon rank‐sum test: *W* = 3950, *p* < 0.001; Figure 6A). Importantly, we found a stark contrast in the distribution of “alive” versus “dead” colony status reports between CS and PM (Pearson's Chi‐square test: *χ* ^2^ = 176, df = 1, *p* < 0.001; Figure 6B), where 76% of CS reports indicated alive colonies compared to 42% in PM. In fact, while only 3% of active colonies in personal monitoring were not reported again the following year, this was the case for 59% of occupied nesting sites in Citizen Science monitoring. Under the simplified assumption that these colonies did not survive, the estimated annual survival rate in Citizen Science monitoring would be just about 13%—closely matching the rates observed through PM.

To assess the effectiveness of the reporting in spring, we quantified the proportion of reports considered “late reports”, defined as submissions after the swarming onset date for that year. The median reporting date after winter for CS was consistently later than for PM, specifically 21 April versus 29 March, respectively (mean: 3 April for PM vs. 2 May for CS) (Figure 6C). Consequently, a substantial proportion of CS reports were rendered unusable annually, i.e., around 46% (84 out of 184) reports of CS could not be used each year, as they were reported too late (compared to 11% in PM, 15 out of 131 reports). These findings highlight an urgent need for improved reporting frequency, intensity and timelines—especially for abandoned nest sites‐ within Citizen Science programs to ensure data reliability.

## 4 Discussion

We launched the BEEtree‐Monitor to investigate the lives and claims on high survival rates of free‐living honeybee colonies in Germany. To do so, we designed a standardized monitoring scheme combining personal monitoring and Citizen Science. This initiative resulted in the most extensive dataset on the topic to our knowledge to date, enabling a comprehensive analysis of the nesting habits and life histories of these colonies over a seven‐year period. It also allowed us to detect and reflect on potential biases in Citizen Science data, which can now be addressed in future projects.

The accumulation of free‐living colonies in Munich and other cities could be attributed to many factors, but is more likely driven by three main reasons. First, higher human population density correlates with higher densities of beekeepers and managed hives, which in turn leads to more escaping swarms searching for cavities (Oré Barrios et al. 2017; von Büren et al. 2019). For example, in Munich, the known density of managed hives exceeds 12 per km^2^, surpassing the German average density by a factor of 4.3 (personal information from the veterinary office and German Ministry of Food and Agriculture 2022). High managed honeybee densities in many European regions have sparked ongoing debates about food competition and pathogen transfer among pollinators (Geldmann and González‐Varo 2018; Alaux et al. 2019; Ropars et al. 2019; Saunders et al. 2018; Herrera 2020; Iwasaki and Hogendoorn 2021; Ghazoul 2005; Casanelles‐Abella and Moretti 2022; Weissmann et al. 2023; Egerer and Kowarik 2020). Such concerns could be particularly pronounced in urban settings where endangered solitary bees are present and the density of managed honeybee colonies is already high (Mallinger et al. 2017; but see Harder and Miksha 2022; Steffan‐Dewenter and Tscharntke 2000). Yet, our findings suggest that the density of free‐living colonies in Munich remains relatively low, at approximately 4% of the density of registered colonies in the city. While the local densities of managed colonies require careful consideration, free‐living colonies should not be considered problematic for urban pollinators nor for managed colonies from urban beekeepers—see Kohl, D'Alvise, et al. (2023) for an investigation on the parasite loads of free‐living colonies.

Second—and perhaps more importantly—the close proximity of honeybee colonies to humans in urban areas increases the likelihood of discovery, potentially skewing perceptions of the distribution and density of free‐living colonies across different habitats. To study the actual distribution, this issue can be addressed by incorporating systematic approaches with random sampling techniques, such as beelining (Seeley 2016; Kohl and Rutschmann 2018; Radcliffe and Seeley 2018; Chakuya et al. 2022).

Third, free‐living honeybee colonies continue to exhibit strong preferences for nesting in tree cavities, echoing their evolutionary history. An interesting aspect is that free‐living honeybees exhibited not only a preference for elevated cavities but also a pronounced directional preference for southern or southwestern nest entrance orientations when selecting cavities. This preference aligns with the thermoregulatory benefits of southern orientations, which facilitate sun exposure and warmth, particularly beneficial during spring when colonies are emerging from winter. However, our data reveal that many of the tree species most frequently occupied by honeybees are now rare in managed forests and are more commonly found in urban or semi‐urban areas. For example, lime trees (*Tilia* spp.)—the most frequently occupied species in our dataset—are of minor importance in modern forests but are abundant in cities and along roads as alley trees. Ash trees (*Fraxinus* spp.), another frequently used nesting species, are also more commonly found in urban environments. However, as they are threatened by *Hymenoscyphus fraxineus*, the causal agent of ash dieback, the proportion of colonies nesting in buildings is likely to increase.

German forests do not provide diverse and rich foraging opportunities throughout the year (Rutschmann et al. 2023), while urban areas offer higher floral diversity, fewer pesticides, and a range of nesting cavities (Ayers and Rehan 2021; Baldock 2020; Young et al. 2021; Garbuzov et al. 2015; Samuelson et al. 2022). Nevertheless, our data show that urban survival rates are not significantly higher than those in forested areas (Kohl et al. 2022). While urban settings may offer foraging and nesting benefits, these do not seem to translate into significantly higher winter survival.

In contrast, regions such as Spain and the UK have reported higher survival rates of free‐living colonies, suggesting potential self‐sustainability (Kohl and Rutschmann 2024; Rutschmann et al. 2022; Visick and Ratnieks 2024). Notably, in Gwynedd, Wales, the use of acaricides in beekeeping is no longer necessary (Valentine and Martin 2023; Remter 2015). Our personal monitoring in Munich, however, indicates that the survival threshold required for self‐sustaining cohorts—approximately one‐third annual survival (Kohl et al. 2022; Rutschmann et al. 2025)—is not being met. This finding aligns with other studies conducted in variable habitats in Germany and Switzerland (Kohl et al. 2022; Lang et al. 2022; Cordillot 2024), yet contrasts sharply with anecdotal claims from lay observers who report continuous and prolonged occupancy of cavities. These observations do not necessarily confirm extended lifespans of individual colonies; rather, it seems more plausible that many free‐living colonies in Germany are recent escapees from managed apiaries, and that monitoring has not been conducted with the necessary rigor.

The factors contributing to the decline of free‐living colonies are varied, encompassing both ecological and evolutionary aspects. From an ecological perspective, challenges such as a shortage of floral resources and lack of suitable and well defendable nesting sites can significantly impact colony viability (Kohl, Rutschmann, et al. 2023; Rutschmann et al. 2023), while parasites, though present, may not play a major role in colony mortality in Germany at the moment (Kohl, D'Alvise et al. 2023; Kohl, Rutschmann, et al. 2023). Another aspect to consider is the evolutionary impact of modern beekeeping practices: They focus on breeding for desirable traits such as maximized honey production, reduced swarming tendencies, and docility (Seeley 2019). Breeding efforts for varroa resistance exist, but they have not yet made a significant impact (Guichard et al. 2023), and the continuous medical treatment of managed colonies prevents tolerance traits from prevailing at the population level (Blacquière et al. 2019; Neumann and Blacquière 2017). Additionally, in Germany, the replacement of the native subspecies *Apis mellifera mellife*ra with non‐native subspecies may have profound implications on the genetic diversity and adaptability of honeybee populations in this area, potentially influencing their ability to thrive independently (Büchler et al. 2014; Dustmann and von der Ohe 1988; Meixner et al. 2015).

Local European honeybee populations consist of deeply entangled cohorts of managed and free‐living colonies in varying proportions. For centuries, the breeding of the former and the natural selection of the latter have impacted the overall population. With the advent of *Varroa destructor* and the continuing use of acaricides in most beekeeping practices, the ratio might have shifted in some European regions, including Germany. We suggest understanding honeybees in Europe as a liminal species, with larger cohorts living under beekeeping conditions and smaller ones living autonomously, both within a broad and shared geographic range. These cohorts are unlikely to be found independently of each other and are deeply interconnected through genetic exchange via mating and annual swarming. Depending on future conservation strategies, populations might either shift towards full dependency on human care or retain and even enhance their capability to self‐sustain by adapting to new environmental circumstances.

In light of our findings, retaining the potential for self‐sustaining honeybee cohorts requires a combination of conservation planning, sustainable beekeeping, and urban planning. Ensuring the availability of suitable nesting sites in forage‐rich and pesticide‐free environments will be essential. Our findings highlight that cavities in buildings have become an important habitat resource for free‐living colonies, particularly as existing tree hollows diminish. This should be considered in urban planning, which could help mitigate potential human‐wildlife conflicts by incorporating designated and low‐conflict nesting structures and public education about species that rely on cavity‐rich urban ecosystems. To ensure that conservation strategies are well‐informed, future Citizen Science efforts should build on insights from this study to improve data accuracy and reduce observational biases.

While personal monitoring (PM) provided structured and repeated observations, the analysis of Citizen Science (CS) data revealed several reporting biases. Many colonies were reported only once, and spring observations were often delayed, leading to data gaps in survival analysis. In our dataset, 76% of CS reports indicated living colonies, compared to only 42% in PM. These patterns suggest that lay observers may report primarily when colonies are active and visible, discontinuing when colonies die or nesting sites vanish. This discrepancy aligns with the 52% and 43% living colonies reported in two other studies, Rutschmann et al. (2022) and Kohl et al. (2022), respectively. Understanding and addressing these biases is essential for accurately interpreting survival rates of free‐living honeybees based on Citizen Science data.

To improve CS data quality, we recommend adopting a standardized monitoring scheme as developed during this study (Figure 1B). We suggest three site visits per year to detect pollen import and to gather empirical data on the swarming season (especially its onset). Citizen scientists need to be reminded early and often enough and briefed on appropriate conduct, timing, and weather conditions for observations. They need to be particularly informed about the excluding relevance of the swarming onset and of reporting unoccupied cavities as rigorously as the occupied ones. In that sense, future Citizen Science monitoring could benefit from designating a subset of nest sites to be monitored jointly by both experts and citizen scientists. During these sessions, observers could be trained to differentiate between various flight patterns, such as foraging, robbing, or scouting, along the criteria offered in this study. Including a commentary section in the monitoring forms proved valuable, as it allowed observers to describe their observations in detail. This additional information helped us to assess the reliability of the data, especially in cases where the data seemed suspicious or lacked information on pollen import. The comparison of PM and CS following the same protocol is a good validation strategy and should be included, and future projects should incorporate early comparisons to identify and address emerging biases.

In essence, structured and standardized monitoring projects are indispensable for thoroughly understanding the mechanisms underlying the survival of free‐living honeybee colonies. Long‐term monitoring and more extensive geographic coverage would enhance our understanding of the survival and reproductive success of free‐living honeybee colonies in Europe. Collaborative efforts by professional researchers and citizen scientists prove beneficial in achieving these objectives. In this sense, occurrence and overwintering survival serve as practical and easily recordable indicators for identifying regions in Europe with high survival rates and larger proportions of free‐living cohorts. Such an approach requires minimal investment in monitoring and does not rely on sophisticated kinship analysis, making it accessible for widespread Citizen Science participation.

## Author Contributions

**Benjamin Rutschmann:** conceptualization (equal), data curation (equal), formal analysis (lead), methodology (equal), resources (supporting), visualization (lead), writing – original draft (lead). **Felix Remter:** conceptualization (equal), data curation (equal), investigation (equal), methodology (equal), project administration (equal), resources (lead), writing – original draft (lead). **Sebastian Roth:** conceptualization (equal), data curation (lead), investigation (lead), methodology (equal), project administration (equal), resources (equal), software (lead), writing – review and editing (equal).

## Ethics Statement

No honeybees were harmed during this study, as we used non‐invasive monitoring methods. Ethical approval was therefore not required.

## Conflicts of Interest

The authors declare no conflicts of interest.

## Supporting information


**Data S1.** Supporting Information.

## Data Availability

Data supporting the findings of this study are publicly available via the Zenodo repository: https://doi.org/10.5281/zenodo.15511556. Personal information of citizen scientists has been excluded to protect participant privacy.
