# Supplementary material for: Monitoring Free‐Living Honeybee Colonies in Germany: Insights Into Habitat Preferences, Survival Rates, and Citizen Science Reliability
Source: Ecol Evol. 2025 Jun 5;15(6):e71469. doi: 10.1002/ece3.71469 (PMC12141091; doi:10.1002/ece3.71469)
Supplement: Supplementary file 1 — Data S1. Supporting Information. [file ECE3-15-e71469-s001.zip › SI_Rutschmann_Remter_Roth2.pdf]

Location of wild colony

It's important to state the location of the find as precisely as possible. You can do this by clicking the map or inputting the information manually into the field below in form of a GPS-coordinate (for example 50.848034,0.23579836). **Please zoom into the map as much as possible** and choose the location of the find (the map allows for an accuracy of apps. 10m). Alternatively, use your smartphone or a GPS device to determine the co-ordinates as accurately as possible. The exact position (using a smartphone) can be found by clicking the compass symbol behind the field of coordinate in this form. **Note:** By clicking on the plus symbol at the top right of the map, you can switch to a different map view. For example, the "Google Hybrid" view offers more details in order to be able to specify the location more precisely.

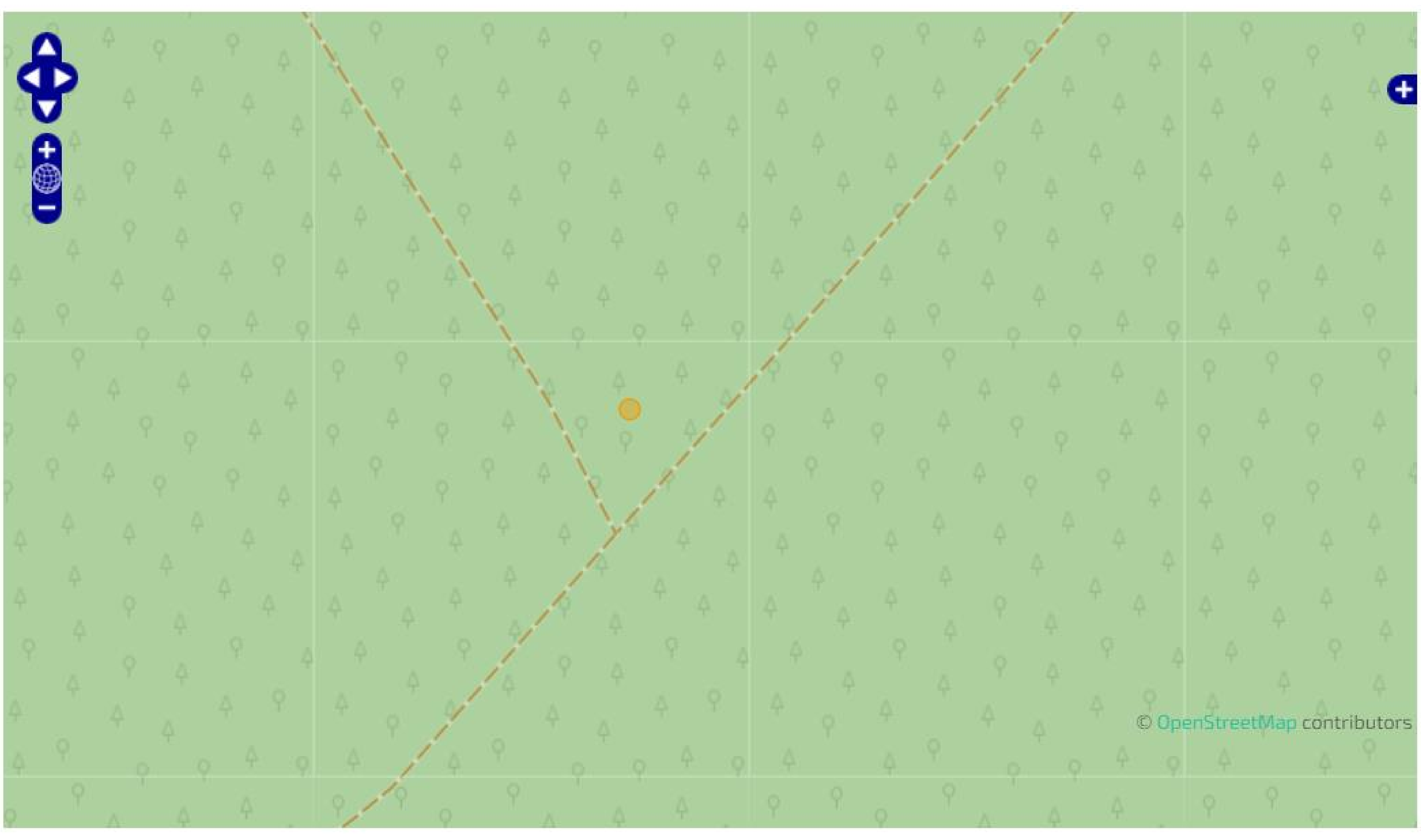

Coordinate: *

Name of location: *

Please give the location an identifiable name in order to easily retrieve it for later additions, for example *Sherwood Forest*, *Victoria Park, London*, *Emerson College, Sussex*.

Description:

Please describe in a few sentences the location of the wild colony, the type of nest (tree, chimney, rock wall, masonry, church eaves, other), state how you became aware of it, and whether there are any problems with the bees you were able to identify.

Date of discovery:

Please click *

Please state the date **when you first saw** bees at this location. This needs to be an **exact date**. If you can't remember you might just estimate the time of year and put in 1st of March if it was Spring, 1st of July if it was Summer or 1st of October for Autumn.

Type of cavity:

Select...

- Select...
- tree cavity
- rock crevice
- building - wall or floor
- chimney
- without cavity - free comb
- other

If the type of cavity/nest site is not listed, please choose "other" and describe it in as much detail as possible in the comment field.

Height of entrance:

Please state the approximate height of the flight hole from the ground **in metres** (for exmaple 7.5 m) - again you can also use the arrows to increase or decrease the number stepwise.

Diameter of entrance hole:

Please state the approximate size of the entrance hole **in centimetres** (for example 6 cm).

Orientation of entrance:

Select...

- Select...
- North
- North-East
- East
- South-East
- South
- South-West
- West
- North-West

Please state the orientation of the flight entrance as South, SE, W, etc..

Shape of flight entrance:

Please describe the form of the entrance, particularly any deviations from the round hole commonly found, e. g. slot-like, longitudinal slot, crevice in rock, oval etc.

Latest Observation

Date of latest observation:

Please click *

Flight activity:

Select... *

- Select...
- intense - more than 25 bees at entrance at the same time
- strong - 10-25 bees at entrance at the same time
- moderate: 1-9 bees at entrance at the same time
- weak - 1-10 bees to be seen at entrance in 10 seconds
- none - even after observing for several minutes
- none - BUT temperatures were maybe too low

Did you recognize bees fly in and out?

Pollen intake:

- ○ no
- ○ yes
- ○ don't know

Clear selection — Did you see **pollen bearing** bees flying into the nest? Binoculars will be helpful for seeing this!

Comments:

Please share any other observations here, such as dead bees found on the ground below the entrance, or if you see other creatures/wasps/birds etc. at the entrance.

Photos

Add photo

Please upload up to three photos. **Don't climb the tree to take these!** Use a tele lens or take the photo with a smartphone through the binoculars. This takes a bit of practice but it works great.

Personal data

Email: *

First name:

Last name: *

Important information:

To make sending the information as simple as possible and encourage continuous input of observations, a user account with an email address will be created when you send this form. This needs to be confirmed (so-called opt-in). To confirm you will receive an email with further instructions.

☐ I would like to be informed by email about important dates (monitoring in spring, summer and autumn) and new website publications, to keep up-to-date and contribute to further research (please note: we will **not** send you any advertising or unrelated communications).

☐ I hereby confirm that I have read the data protection statement of BEEtree-Monitor and that I am in agreement with these.*

Submit
