# Supplementary material for: Monitoring Free‐Living Honeybee Colonies in Germany: Insights Into Habitat Preferences, Survival Rates, and Citizen Science Reliability
Source: Ecol Evol. 2025 Jun 5;15(6):e71469. doi: 10.1002/ece3.71469 (PMC12141091; doi:10.1002/ece3.71469)
Supplement: Supplementary file 1 — Data S1. Supporting Information. [file ECE3-15-e71469-s001.zip › SI_Rutschmann_Remter_Roth.pdf]

# SUPPORTING INFORMATION

Acknowledgment appendix

We thank Andrea Voit, Andreas Schmitt, Andrej Barth, Anja Salg, Anna Dünser, Antonio Gurliaccio, C. Hutschenreuther, Christian Ahrens, Claudia Schneider-Ludwig, Frank Schuberdt, Frank Soukup, Gottfried Schumann, Hagen Morscheck, Hannes Oberreiter, Ingo Schüler, Jens Eickmeier V.N., Kai Oliver Siegenthaler, M. Billmeier, Markus Kimpel, Martin Dannenberg, Michael Ostheer, Moses Martin Mrohs, Olivia Ortlieb, Omar Alejandro Machado Taboada, Peter Pfanzelt, Sergej Hübert, Sven Büchner, Thomas Mull, Thomas Schneider-Ludwig, Vivien Otto, W. Schwarz, Waldemar Schmidt and Wolfgang Mück and all the rest of the BEEtree-Monitor community. We would like to express our gratitude to the HOBOS Team of the University of Würzburg for their support with the BEEtrees project during the initial stages of our research. Special thanks go to Jürgen Tautz, Marina Kretzschmar, Gerhard Vonend, Kristina Vonend, Anneli Kiessling, Konrad Öchsner, Hartmut Vierle and the rest of the team. We thank the Munich Centre for Technology in Society (MCTS) at the Technical University of Munich and the Centre for Urban Nature and Climate Adaptation (ZSK) for supporting the BEEtree project during the research process. Thanks go to Regine Keller and Ignacio Farías. We also thank André Wermelinger, Uwe Lang, Frank Krumm and Patrick Kohl for their valuable input during the initial discussions on the monitoring protocol. Additionally, we acknowledge Valerie Kantelberg, Sigrun Mittl, Andreas Schierling and Julie Weissmann for their insightful discussions on the topic.

Platform and dataset

While participants were initially asked to fill in a designated form and send it to us via email, a dedicated platform was developed later on. The platform was created entirely from open-

source software. We utilized a Debian Linux server as the foundation. Furthermore, we employed the classic combination of the Apache web server, PHP and PostgreSQL database, extended with Drupal and Indicia (the Open-Source Wildlife Recording Toolkit).

To maintain confidentiality, all reporting information was anonymized for evaluation and analysis, ensuring that inference to individual persons and colonies was not possible. The dataset of the BEEtree-Monitor used for this publication is as of August 2023. Survival data pre winter 2016/2017 were discarded due to small sample sizes.

Grouping of CORINE Land Cover map classes

To classify the nesting and foraging habitats of the reported free-living colonies, we utilized the land cover classes from the CORINE Land Cover map 2018 (European Environment Agency, 2018) and grouped them into five major categories based on land use and habitat characteristics. Specifically, **urban** included 'discontinuous urban fabric', 'continuous urban fabric', 'industrial or commercial units', 'green urban areas', and 'sport and leisure facilities'; **cropland** comprised 'non-irrigated arable land', 'vineyards', 'complex cultivation patterns', 'fruit trees and berry plantations', and 'land principally occupied by agriculture, with significant areas of natural vegetation'; **grassland** encompassed 'pastures'; **deciduous forest** included 'broad-leaved forest', 'mixed forest', and 'peat bogs'; and **coniferous forest** consisted of 'coniferous forest'. For certain analyses, these five categories were further consolidated into three main groups to facilitate statistical comparisons: **Urban** (urban areas), **rural** (cropland and grassland), and **forest** (deciduous and coniferous forests). Water bodies and water courses were excluded from the grouping.

Annual survival rates vs. seasonal survival rates and reanalysis of datasets

Some studies calculate seasonal survival rates, while others present annual survival rates. Both approaches have advantages and disadvantages. Seasonal survival rates are well-suited

for expert-based monitoring schemes where nest sites are visited at defined time points during the year, e.g., three times as in this project in Munich or in Kohl et al. 2022). In contrast, citizen science reports often have fluctuating response rates, leading to some nest sites lacking reports. Therefore, it might be reasonable to include nest sites where monitoring was not always conducted using a strict approach. As explained in the main text, we used the following rationale:

"We defined survival as the instance of a cavity being occupied from summer (during or after the swarming season) until the following spring, before the next swarming season commenced (Figure 1B). One valid report of an active colony in spring before the start of the swarming season (1st check) is proof of overwintering survival. Additionally, we implemented and encouraged participants to conduct one or two more annual checks: after the end of swarming (2nd check) and in Autumn (3rd check). Post-swarming checks at all known cavities (including the recently unoccupied ones) served to find the new founder colonies for further monitoring, while the Autumn check (3rd check) detects summer deaths (we attributed these as perished to the survival statistics of the following year)."

Our study did not account for the possibility that the death of a colony in spring is followed by the quick re-occupation of the cavity by a new swarm without being noticed. The rate of such "silent spring turnovers" was reported to be 11.1% (Kohl et al. 2022). Therefore, our annual survival rate might be slightly overestimated.

The published numbers of colonies reported in Kohl et al. (2022) and Lang et al. (2022) slightly differ from the ones presented here, as we reanalyzed the survival of each colony reported in Kohl et al. (2022) and Lang et al. (2022) with the yearly swarming onset observed for this study in each colony. Survival observations in these two studies after the onset of swarming were discarded as not reliable indicators of survival.

Model selection

To ascertain the influence of various predictors including monitoring types and year on the odds of colony survival, we compared several mixed-effects logistic regression models. We initially tested an interaction model on our dataset (PM and CS) to explore whether the effect of monitoring type varied across time, using the formula *survival ~ type* × *year_grouped + (1 | id)*, where years were grouped into early monitoring years (≤2020) and late periods (2021-2023). However, the interaction term was not significant ($p = 0.801$), indicating that the effect of monitoring type on survival rates did not change over time. For the dataset including the additional studies (Kohl et al. 2022 and Lang et al. 2022) the following models were tested:

- Model 1 included 'monitoring type' as a fixed effect (with the factor levels: CS, PM, Kohl et al. 2022 and Lang et al. 2022) and 'colony id' nested within 'year' as a random effect to account for repeated measures on the same colonies across years.
- Model 2 included both 'monitoring type' and 'year' (from 2016 till 2023) as fixed effects, with 'colony id' as a random effect.

Model selection based on AIC values favoured Model 2.

Exemplary estimation of free-living honeybee colony density in Munich

Using QGIS, we defined the study area in Munich by generating a polygon that encapsulated 92 known nesting sites as of fall 2023, covering an area of 160 km² (Figure SI1). This enabled us to calculate the density of nest site locations of 0.58 cavities/km² within this defined boundary (polygon, Figure SI2). This nest site location density was considered to be constant across all study years, despite the gradual addition of new nesting sites over the years. Thereafter we calculated an occupancy ratio of the known 92 cavities based on reported cavity statuses ("alive" or "dead") for three distinct seasonal periods: in spring prior

to the start of the swarming season (around 15 April), in summer after the swarming season (around 15 July) and in fall (around the 1 October).

Only nesting locations with status reports for a given time point were included in the calculations. The highest number of cavities with a reported colony status in Munich occurred in the summer of 2020 with 66 reports. During the observed spring seasons from 2018 to 2023, occupation rates were consistently low, with no occupation of the vacant nest sites reported in spring 2019 and a peak of 29.4% in spring 2018 (mean: 10.5%). In contrast, summer seasons exhibited generally high occupation rates, with a peak of 86.1% in summer 2018 and the lowest rate of 60.0% in summer 2021 (mean: 72.3%). The average occupation rate in the fall was 48.7%.

To estimate the minimum density of free-living colonies, we multiplied the constant nest site density by the calculated occupancy ratio for each year and designated seasonal time point (see Figure SI3 and Figure SI4). This provided us with a temporal minimum density estimate for free-living honeybee colonies in Munich (0.06 colonies per square kilometer in spring, 0.42 in summer, and 0.28 in fall).

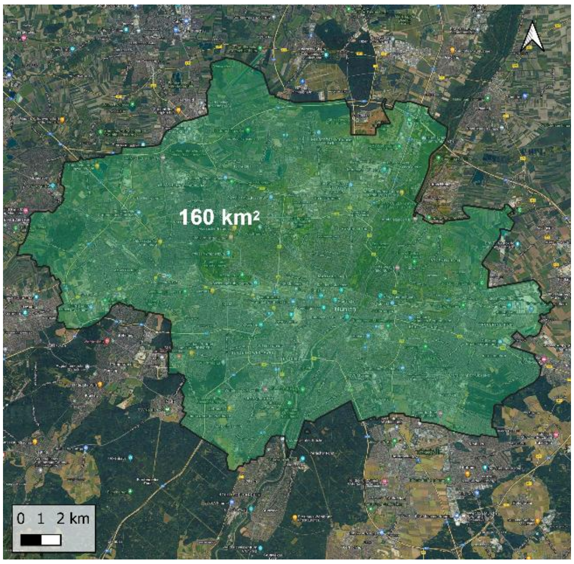


*Figure SI1: Polygon of the Kreisfreie Stadt Munich for calculating the study area.*

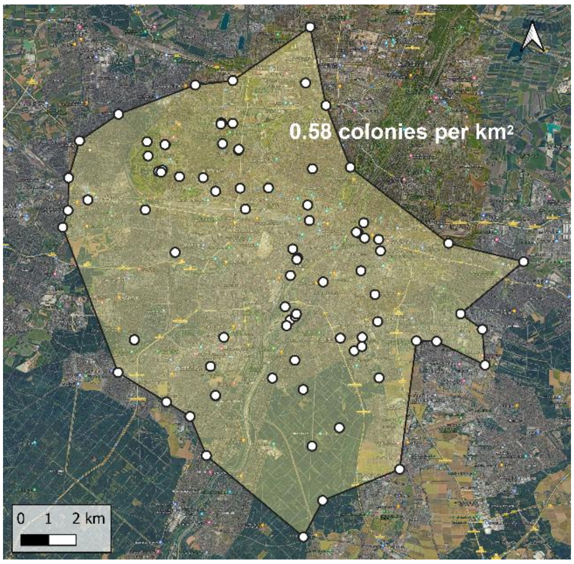


*Figure SI2: Distribution of nest sites of free-living honeybee colonies to calculate the nest site density in the city of Munich.*

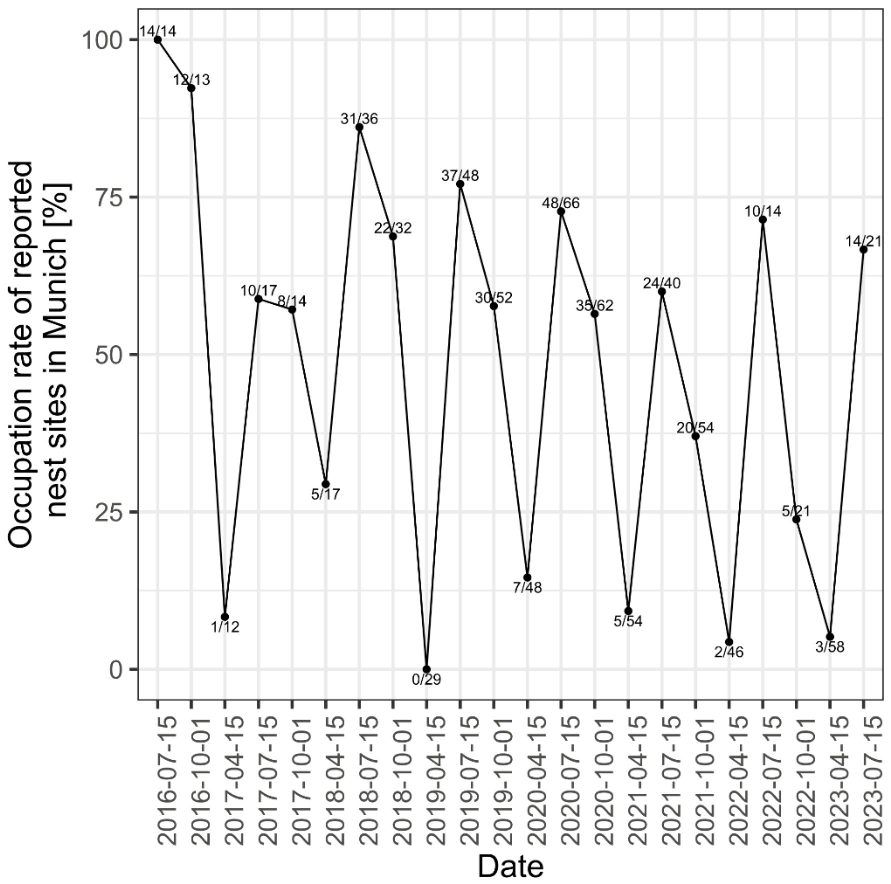


*Figure SI3: Occupation rate of nest site locations in Munich across the different seasons and years. The numbers in the plot give the ratio of active to total nest site reports.*

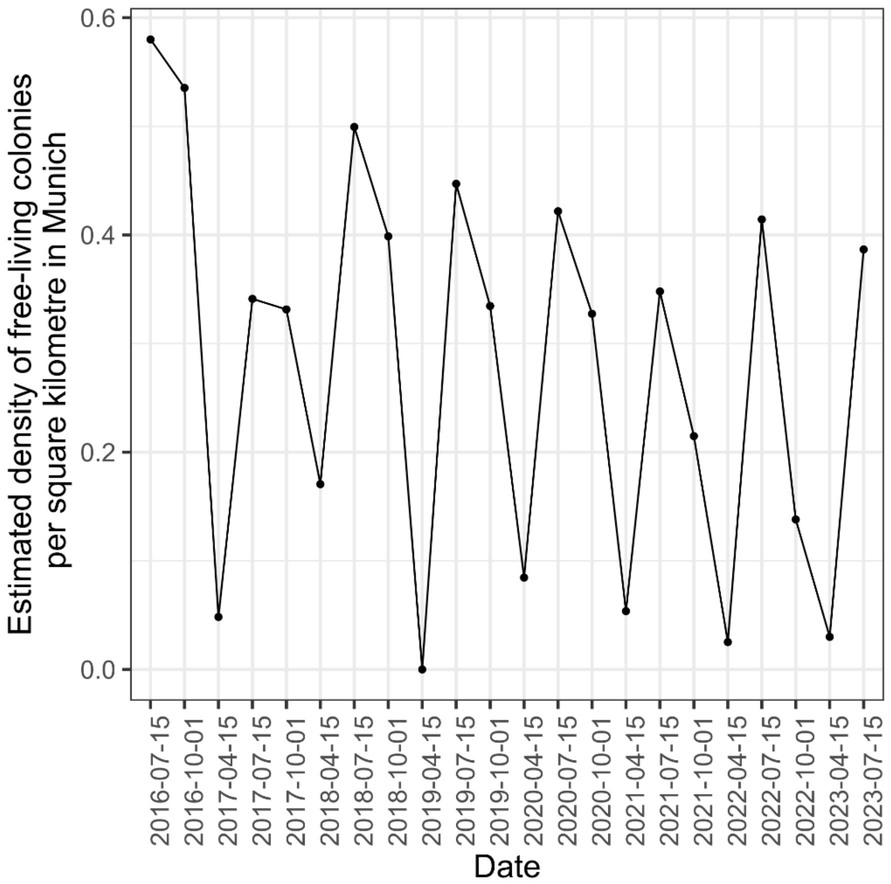


*Figure SI4: Estimated density of free-living colonies per square kilometer in Munich across the different seasons and years.*

Distribution of nesting and foraging habitat of reported free-living honeybee colonies

To compare the distribution of free-living honeybee colonies across different habitats with the overall distribution of land cover types in Germany, we performed chi-square goodness-of-fit tests to assess whether the observed distribution of colonies significantly deviates from what would be expected based on the land cover proportions. Two sets of observed habitat distributions were analyzed: the nesting habitats of colonies (see main text) and their available foraging habitats (within 2km of the colony; see Figure SI5). The expected frequencies were based on the proportion of land cover types in Germany, as reported by CORINE Land Cover map classes.

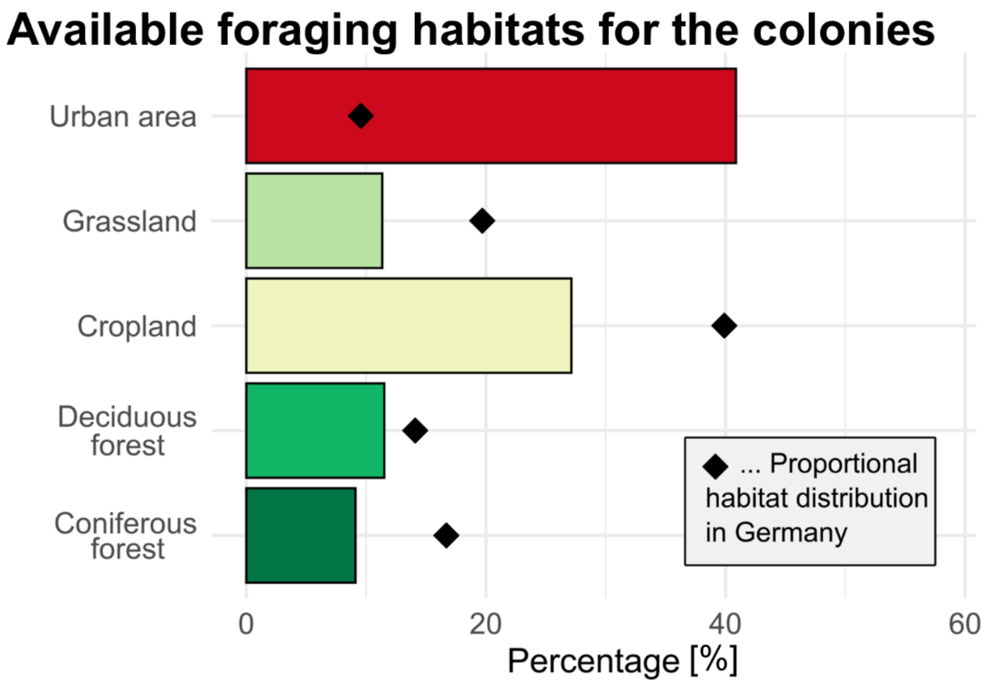


*Figure SI5: Proportional distribution of foraging habitats that is available to the bees (habitat within a 2 km radius around each colony). Black diamond symbols show the relative distribution of a land cover types across Germany.*

<u>Number of observations per colony, reported colony status proportions, onset of swarming and spring reports</u>

In order to compare the number of observations per colony between our personal monitoring (PM) and Citizen Science efforts (CS), we utilized a Wilcoxon rank-sum test as the data did not meet the assumptions of normality and homogeneity of variances. To assess whether the proportion of colonies reported as "alive" and "dead" differed between Citizen Science initiatives and personal monitoring efforts, a chi-square test for independence was employed.

We evaluated the timelines of reports from Citizen Science and personal monitoring, with a particular focus on reports submitted after winter. This period, just before the onset of the swarming season, is crucial for assessing colony survival over winter. Our analysis was based on nest site locations that were active during the previous summer or fall and were reported as alive in the subsequent year. For these colonies, we looked at data on the overwintering reports (starting from March on). The critical part of this analysis was determining whether

these overwintering reports were submitted before or after the start of the swarming season. To assess the effectiveness of the reporting, we quantified the proportion of reports considered 'late reports', defined as submissions after the swarming start date for that year.
